# Heat as an Oxytocic

**Published:** 1885-09

**Authors:** 


### Heat as an Oxytocic.

Dr. W. B. Asbury, of Wotasulga, Ala., reports his experience with heat as an oxytocic, having used it in several cases of tardy labor pains. Hot poultices applied over the fundus uteri invariably were followed by strong regular pains. The poultices are to be hot as the patient can bear them; not simply warm. They are not to be placed down over the pubes, but over the fundus uteri, and changed frequently. He desires the profession to use them and report their success.

